# Supplementary material for: Competing cognitive pressures on human exploration in the absence of trade-off with exploitation
Source: Nat Commun. 2026 Feb 12;17:883. doi: 10.1038/s41467-026-68639-2 (PMC12901306; doi:10.1038/s41467-026-68639-2)
Supplement: Supplementary file 1 — Supplementary information file [file 41467_2026_68639_MOESM1_ESM.pdf]

Supplementary information

Competing cognitive pressures on human exploration in the absence of trade-off with exploitation

Clémence Alméras[1,2,*], Valerian Chambon[2,3], and Valentin Wyart[1,2,*]

[1] Laboratoire de Neurosciences Cognitives et Computationnelles, Institut National de la Santé et de la Recherche Médicale (Inserm), Paris, France

[2] Département d’Études Cognitives, École Normale Supérieure, Université PSL, Paris, France

[3] Institut Jean Nicod, Centre National de la Recherche Scientifique (CNRS), Paris, France

[*] Corresponding authors: clemence.almeras@gmail.com, valentin.wyart@inserm.fr

Contents

Supplementary Discussion page 2

Supplementary Figures 1-12 pages 3-15

Supplementary Tables 1-6 pages 16-20

Supplementary References page 21

Supplementary Discussion

In the following section, we discuss three alternative explanations for the differences we document in exploration patterns in the different conditions, which we want to rule out.

The evidence-dependent repetitive sampling in the beginning of sequences is not reducible to mere perseveration. While previous studies have shown that policy perseveration could arise from memory limitations leading to policy simplification[1,2], the initial repetitions we describe are hardly a simplification of the policy. Repetitions here are not blind: participants only repeat initial choices as long as the level of evidence acquired on the preceding choices is below a certain threshold. In other words, this conditional policy requires to compare the current level of evidence for the option to the threshold level (parameter θ in the model), and based on this comparison, to decide whether to repeat or to compute the decision variable. As such, initial repetitions are a more complex policy than simply always computing the decision variable. Incidentally, in this task, participants are only choosing between two options, which restrains the complexity of potential policies, and makes it unlikely that policy compression be the prime driver of the smart repetition strategies we observed. We argue that repetitive sampling might be easier for humans because it curtails a cognitive limitation linked to the cost of switching. However, while it appears behaviourally simple, it does not necessarily reflect simpler *policies*: it might be more efficient for humans to adopt seemingly more complex policies if they provide evidence in a more cognitively relevant way.

Initial repetitive sampling could aim at first generating hypotheses about each option, before testing them in subsequent choices. Such hypothesis testing strategies have been argued to be more cognitively efficient in self-directed learning contexts because it provides evidence perceived as more relevant at each choice[3,4]. However, this is not to be confused with stickiness in the sampling of hypotheses. Previous studies of categorisation or concept learning have shown that when participants are gradually presented with evidence, they tend to stick to the same hypothesis until it is critically disconfirmed (e.g. [5–7]). But stickiness about the *hypotheses* doesn't explain why participants would want to resample the same *option*. It is very well possible that our participants were also conservative about hypotheses and only changed their minds in the face of sufficiently contradictory evidence, but our results also suggest that they wanted to resample the same *option* until they were sufficiently convinced of their current hypothesis – i.e. until they reached a certain level of evidence in favour of said hypothesis.

Another alternative explanation for the difference in sampling patterns between conditions is to propose that participants relied on different heuristics because the instructions gave them different goals. One could propose that participants repeated choices in the delayed rewards (GUESS) condition because they were free to study each option in isolation, whereas in the MATCH and FIND conditions they were pushed to compare options by sampling one after the other, juxtaposing pairs of outcomes. We did not find any evidence that participants tended to alternate between options in the MATCH and FIND sequences (Supplementary Figure 10). Rather, repetition biases were positive (meaning that participants did not tend to alternate between options in any condition), comparable and correlated across all three conditions (Figure 7.a. and Supplementary Figure 7.b.), and so was the sensitivity to uncertainty across the GUESS and FIND conditions (Figure 7.a.). Given such consistency in parameter estimates across conditions, it is very unlikely that participants resorted to wildly different heuristics in the different conditions. In addition, in the MATCH condition, even in the initial choices, participants' decisions to switch option appeared to depend mainly on the reward value of the preceding outcome (Supplementary Figure 10), rather than demonstrating systematic alternation.

Overall, these alternative explanations cannot account for the pattern of human findings across the MATCH, FIND and GUESS conditions that we report in the main text.

Supplementary Figures

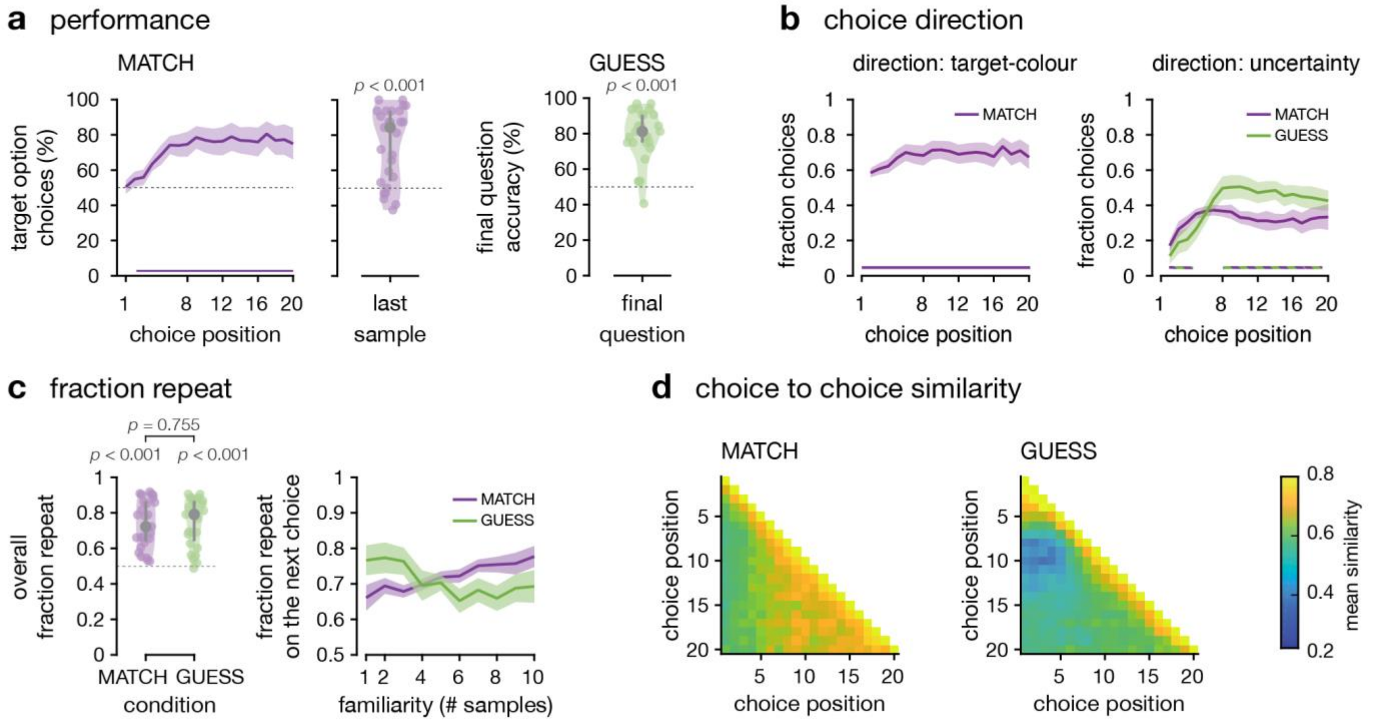

Supplementary Figure 1. Behavioural indices in the confirmatory dataset. *N* = 27 participants, MATCH condition in purple, GUESS condition in green. **a. Performance.** Left: The fraction of choices towards the option truly associated to the target colour is plotted against choice position for single-target sequences (group means ± 95% CI, *N* = 27 participants). Horizontal bar are clusters of significant two-sided *t*-tests against chance, the cluster was then tested against clusters appearing from random choices (see Methods). Center: Fraction of choices towards the option truly associated to the target colour at the last sampling decision of the sequence (8th, 12th, 16th or 20th trial - medians ± inter-quartile ranges, *N* = 27 participants, two-sided signed-rank test against chance - 50%). NB: participants did not know this was the last choice of the sequence. Right: Fraction of correct responses to the final question in GUESS sequences (medians ± inter-quartile ranges, *N* = 27 participants, two-sided signed-rank test against chance (50%). **b. Choice direction.** Left: in the MATCH condition, fraction of target-directed choices at each choice in the sequence (group means ± 95% CI, *N* = 27 participants). For each choice, we accumulated the outcomes observed for each option based on the choices of the participant up to this point in the sequence, and defined the target option as the option with highest accumulated value in direction of the target colour category. Horizontal bar are clusters of significant two-sided *t*-tests against chance, the cluster was then tested against clusters appearing from random choices (see Methods). Right: in both conditions, fraction of uncertainty-directed choices at each choice in the sequence (group means ± 95% CI, *N* = 27 participants). For each choice, we accumulated the outcomes observed for each option based on the choices of the participant up to this point in the sequence, and defined the uncertain option as the option with lowest absolute accumulated value. Horizontal bar are clusters of significant two-sided paired *t*-tests between conditions, the difference was then tested against random permutations of the condition labels (see Methods). **c. Fraction repeat.** Left: mean fraction of repeat decisions in the sequences (medians ± inter-quartile ranges, *N* = 27 participants, two-sided signed-rank test against 0.5 and between conditions). Right: fraction of repeat decisions in the confirmatory dataset, as a function of how many times they had already sampled this option earlier in the sequence (group means ± 95% within-subject CI, *N* = 27 participants). *n.s.*: not significant. **d. Choice to choice similarity.** For each condition, average similarity of each choice to the other choices in the same sequence. Each choice of a sequence was compared to each of the other choices in the sequence, resulting in a similarity score for each pair of choice positions within each sequence. This was averaged across sequences and participants within conditions, resulting in an average similarity score (between 0 and 1) for each position in the sequence in each condition (*N* = 27 participants, 64 sequences per participant per condition).

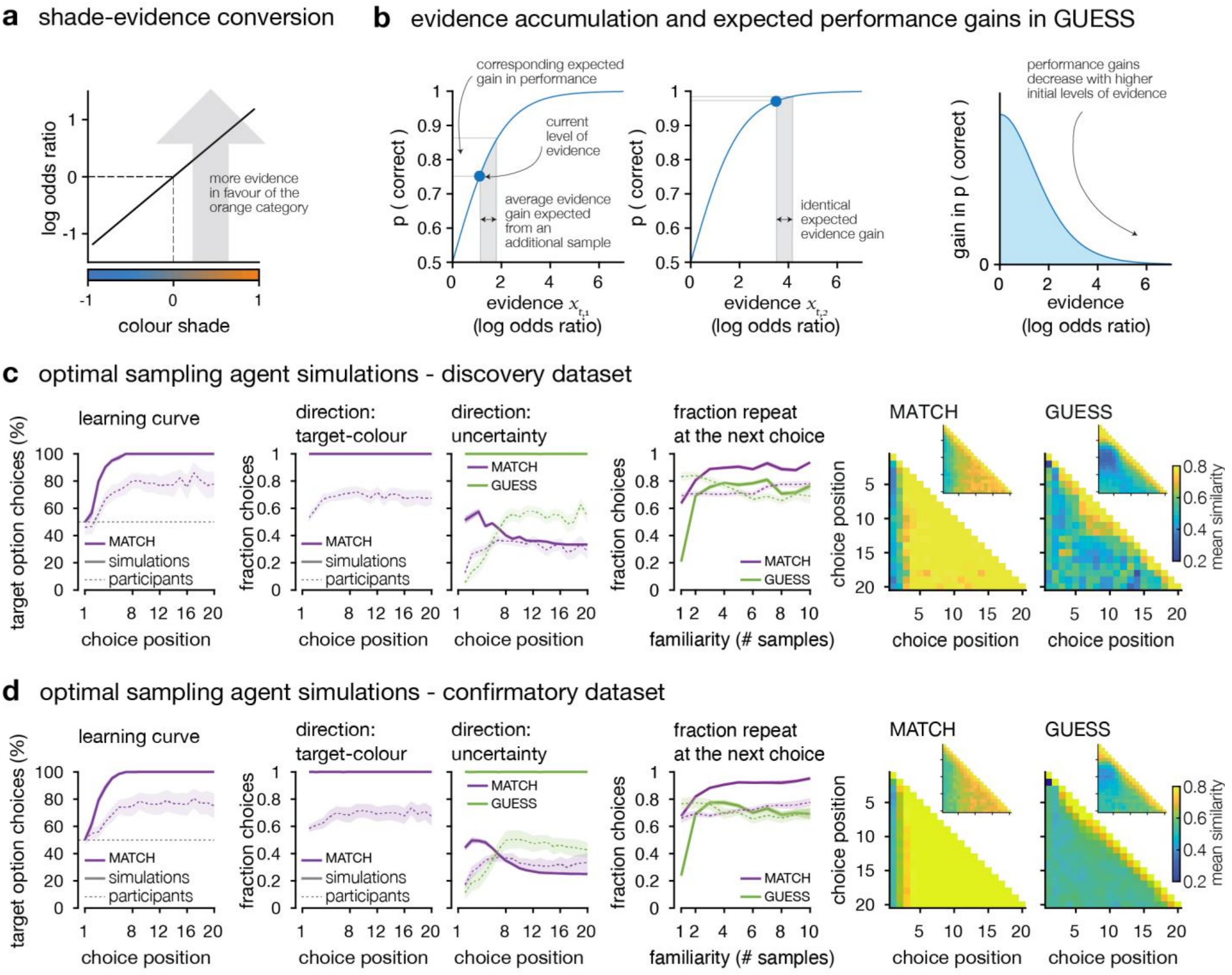

Supplementary Figure 2. Optimal model. a. Shade-evidence conversion. Outcome colours were drawn from a continuum ranging from blue to orange such that the shades were linearly related to the log odds ratio of the categories (blue-dominant or orange-dominant). **b. Evidence accumulation and expected performance gains in GUESS.** At any sampling decision during the sequence, the gain in evidence to be expected from drawing an additional sample was the same. However, the corresponding the *gain* in performance (the probability of giving the correct response at the final question) depends on the current level of evidence: An additional sample is expected to increase performance more when the agent knows little about the option (lower level of evidence, left curve) than when the agent already has a lot of information about the option (center curve). The derivative of the performance curve as a function of the evidence (rightmost curve) shows the decreasing benefit of taking additional samples for a constant expected gain in information. **c. Optimal sampling agent simulations in the discovery dataset.** We simulated a sampling agent with optimal sampling parameters values: $\beta_{\text{tar}} \to \infty$, $\beta_{\text{unc}} \to \infty$, $b_{\text{rep}} = 0$, and no initial sampling phase (*n* = 1000). This agent played the sequences available in the discovery dataset and their choices were processed through the same pipeline as the choices of participants: learning curves, choice direction, fraction of repeat decisions and choice-to-choice similarity (cf. Figure 2). In purple for the MATCH condition, in green for the GUESS condition, simulations in solid lines, participants in dotted lines for reference (group means ± 95%CI). **d. Optimal sampling agent simulations in the confirmatory dataset.** We simulated a sampling agent with optimal sampling parameters values: $\beta_{\text{tar}} \to \infty$, $\beta_{\text{unc}} \to \infty$, $b_{\text{rep}} = 0$, and no initial sampling phase (*n* = 1000). This agent played the sequences available in the confirmatory dataset and their choices were processed through the same pipeline as the choices of participants: learning curves, choice direction, fraction of repeat decisions and choice-to-choice similarity (cf. Figure 2). In purple for the MATCH condition, in green for the GUESS condition, simulations in solid lines, participants in dotted lines for reference (group means ± 95%CI).

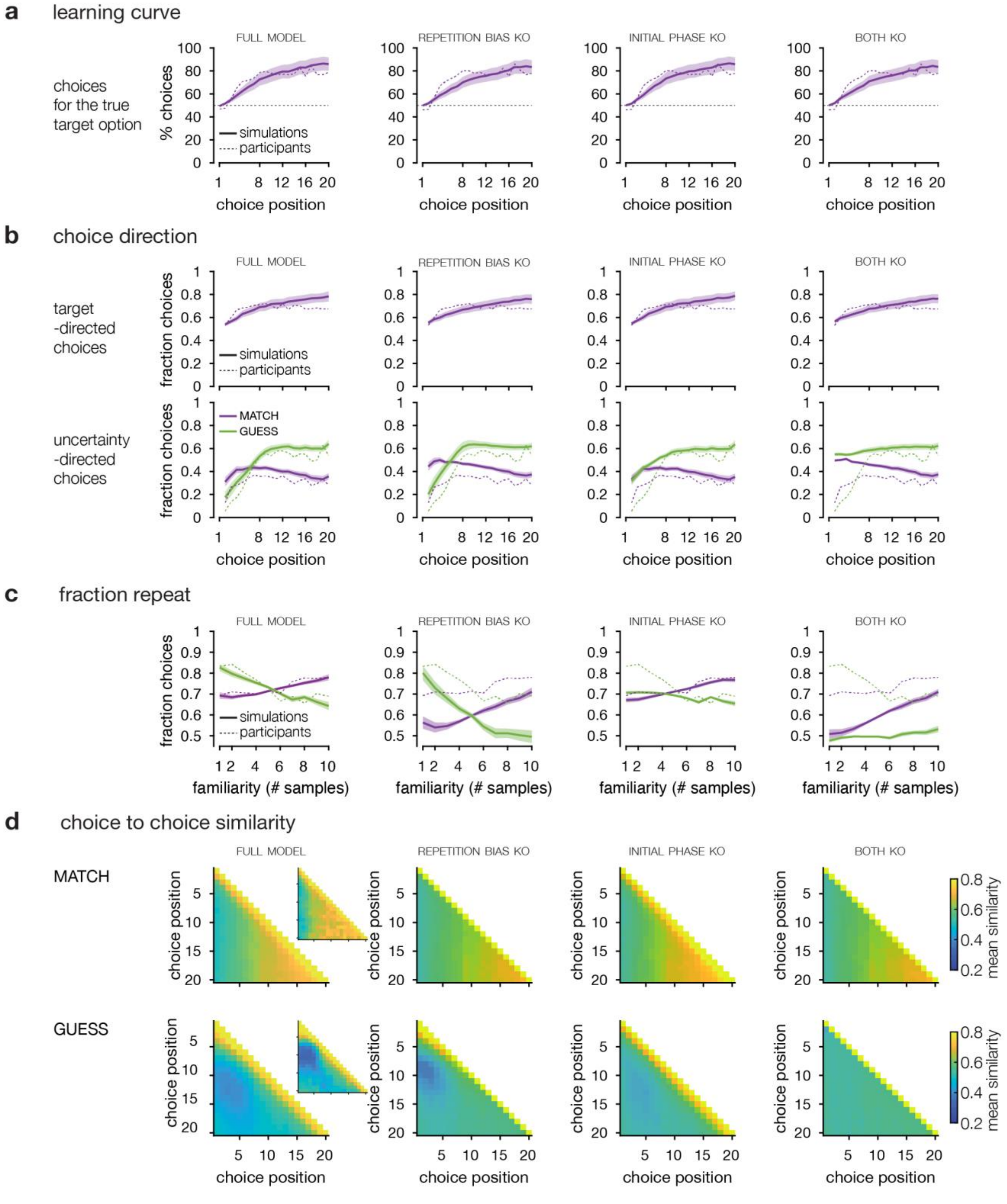

Supplementary Figure 3. Knock-out simulations in the discovery dataset (*N* = 27 participants). Columns displays different simulated 'knock-out' versions of the model: the full version of the model, with sensitivity to the target and to uncertainty, repetition bias and an initial sampling phase (first column), a model without the repetition bias (second column), a model without the initial repetitive phase (third column), and a model without initial sampling phase nor repetition bias, featuring only sensitivity to the target colour and to the uncertainty of options (last column). Choices of the different versions of the model were analysed through the same pipeline as the participants' choices. In purple for the MATCH condition, in green for the GUESS condition. **a. Learning curve.** Fraction of choices towards the option truly associated to the target colour, plotted against choice position for single-target sequences. Model simulations (solid lines, group means ± 95% CI), against participants' average behaviour (dotted lines). **b. Choice direction.** Top: in the MATCH condition, fraction of target-directed choices at each choice in the sequence (solid lines: average of model simulations ± 95% CI, dotted lines: participants' average). For each choice, we defined the target option as the option with highest accumulated value in direction of the target colour category. Bottom: in both conditions, fraction of uncertainty-directed choices at each choice in the sequence (solid lines: average of model simulations ± 95% CI, dashed lines: participants' average). For each choice, we defined the uncertain option as the option with lowest absolute accumulated value. **c. Fraction repeat.** Fraction of repeat decisions on the following choice, as a function of how many times participants had already sampled this option earlier in the sequence (solid lines: simulations' average ± 95% within simulation CI, dashed lines: participants' average). **d. Choice to choice similarity.** For each condition, average similarity of each choice to the other choices in the same sequence, for model simulations (main triangles) and for participants (inlays).

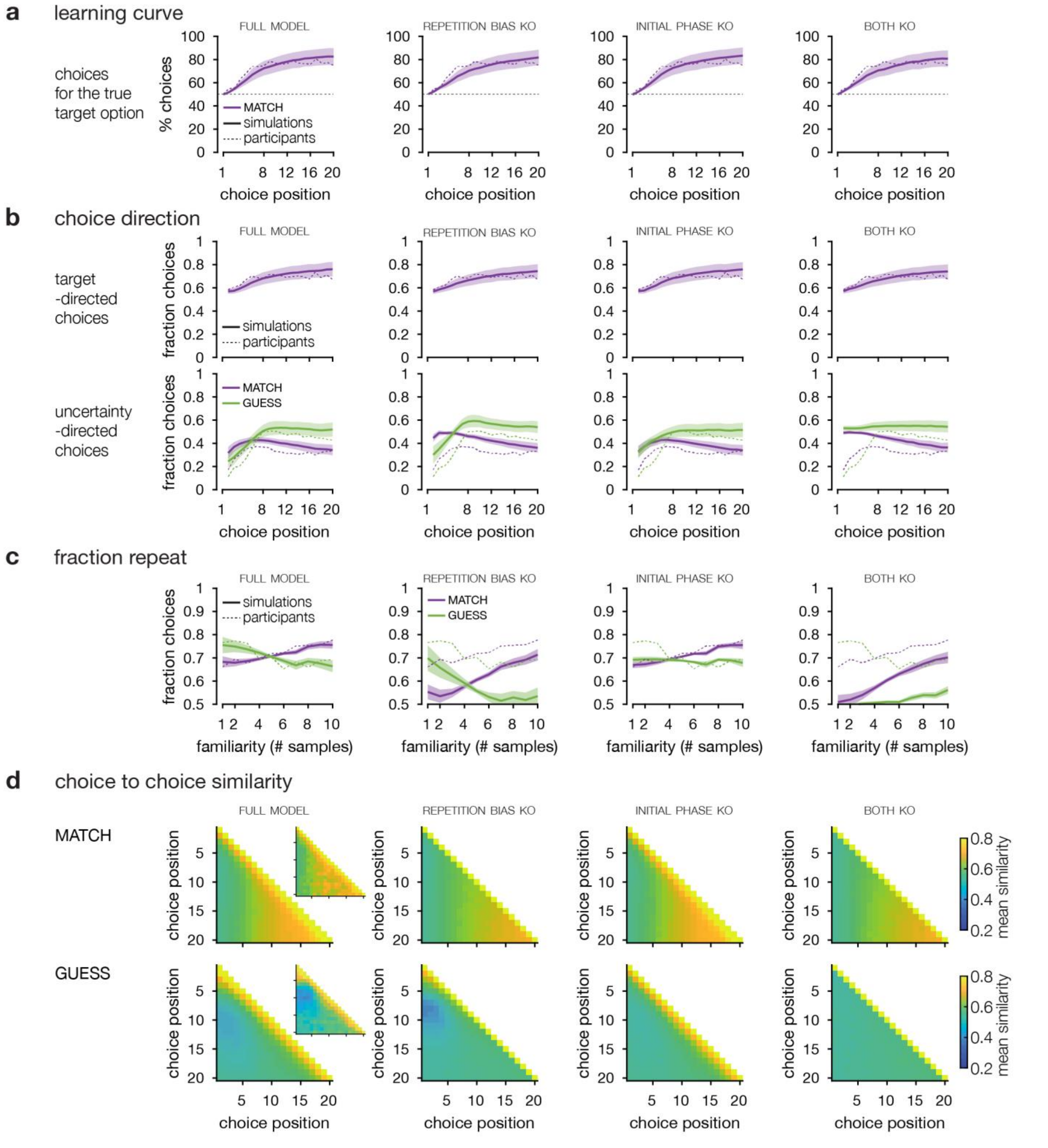

Supplementary Figure 4. Knock-out simulations in the confirmatory dataset (*N* = 27 participants). Columns display different simulated 'knock-out' versions of the model: the full version of the model, with sensitivity to the target and to uncertainty, repetition bias and an initial sampling phase (first column), a model without the repetition bias (second column), a model without the initial repetitive phase (third column), and a model without initial sampling phase nor repetition bias, featuring only sensitivity to the target colour and to the uncertainty of options (last column). Choices of the different versions of the model were analysed through the same pipeline as the participants' choices. In purple for the MATCH condition, in green for the GUESS condition. **a. Learning curve.** Fraction of choices towards the option truly associated to the target colour, plotted against choice position for single-target sequences. Model simulations (solid lines, group means ± 95% CI), against participants' behaviour (dotted lines). **b. Choice direction.** Top: in the MATCH condition, fraction of target-directed choices at each choice in the sequence (solid lines: average of model simulations ± 95% CI, dotted lines: participants' average). For each choice, we defined the target option as the option with highest accumulated value in direction of the target colour category. Bottom: in both conditions, fraction of uncertainty-directed choices at each choice in the sequence (solid lines: average of model simulations ± 95% CI, dashed lines: participants' average). For each choice, we defined the uncertain option as the option with lowest absolute accumulated value. **c. Fraction repeat.** Fraction of repeat decisions on the following choice, as a function of how many times participants had already sampled this option earlier in the sequence (solid lines: simulations' average ± 95% within simulation CI, dotted lines: participants' average). **d. Choice to choice similarity.** For each condition, average similarity of each choice to the other choices in the same sequence, for model simulations (main triangles) and participants (inlays).

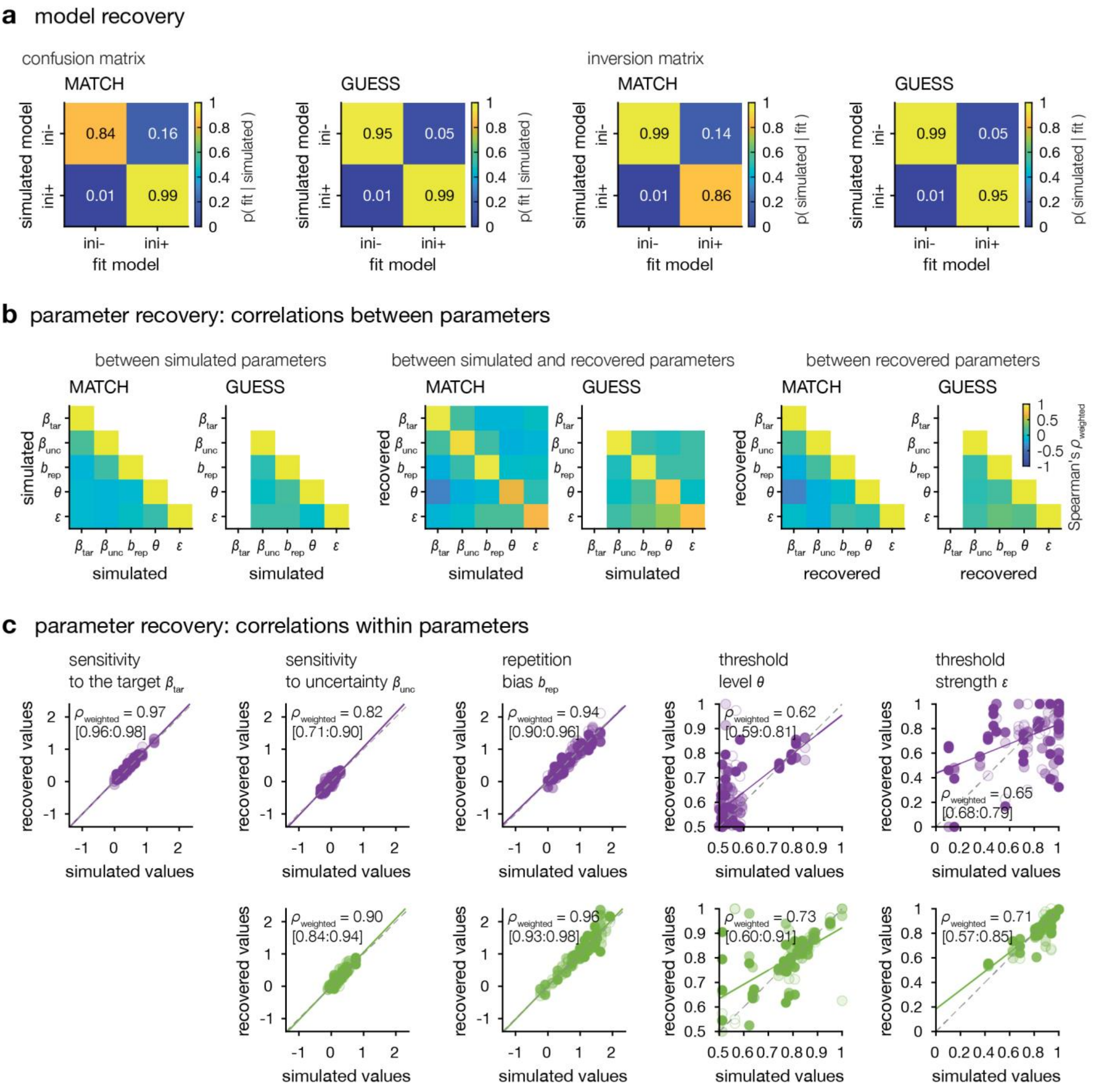

Supplementary Figure 5. Recovery analysis in the discovery dataset. a. Model recovery. Left, confusion matrix: estimated prevalence of a model fit with (ini+) *vs*. without (ini-) initial sampling phase (in columns), for data actually simulated with (ini+) *vs*. without (ini-) the initial phase (in rows). We simulated models using the average of participants' estimates for each parameter in each condition, except for the initial phase parameters for which we simulated both conditions using the average of participants' estimates in the GUESS condition. Right, inversion matrix: probability of data simulated with the ini+ or ini- models (in rows), under each of the ini+ vs. ini- models (in the columns). **b. Parameter recovery: correlations between parameters.** We first permuted the values of each best-fitting parameter to remove between parameter correlations while preserving parameter distributions. We simulated choices using the de-correlated best-fitting parameter estimates for each participant, and then fit the simulated choices using the same procedure as we used on participants' choices (recovery). Left: correlations between the simulated parameter values (Spearman correlations weighted by the goodness of the original fit). Centre: correlations between simulated and recovered (re-fit) values (Spearman correlations weighted by the goodness of the original fit). Right: correlations between recovered (re-fit) values (Spearman correlations weighted by the goodness of the original fit). **c. Parameter recovery: correlations within parameters.** We first permuted the values of each best-fitting parameter to remove between parameter correlations while preserving parameter distributions. We simulated choices using the de-correlated best-fitting parameter estimates for each participant, and then fit the simulated choices using the same procedure as we used on participants' choices (recovery). Recovered values (estimates obtained from the simulated choices) are plotted against original simulated values (participants' best fitting values, obtained from participants' own choices) - Spearman correlations weighted by the goodness of the original fit (opacity level). Top row, in purple for the MATCH condition, bottom row in green for the GUESS condition, N = 108 (27 participants * 4 simulations).

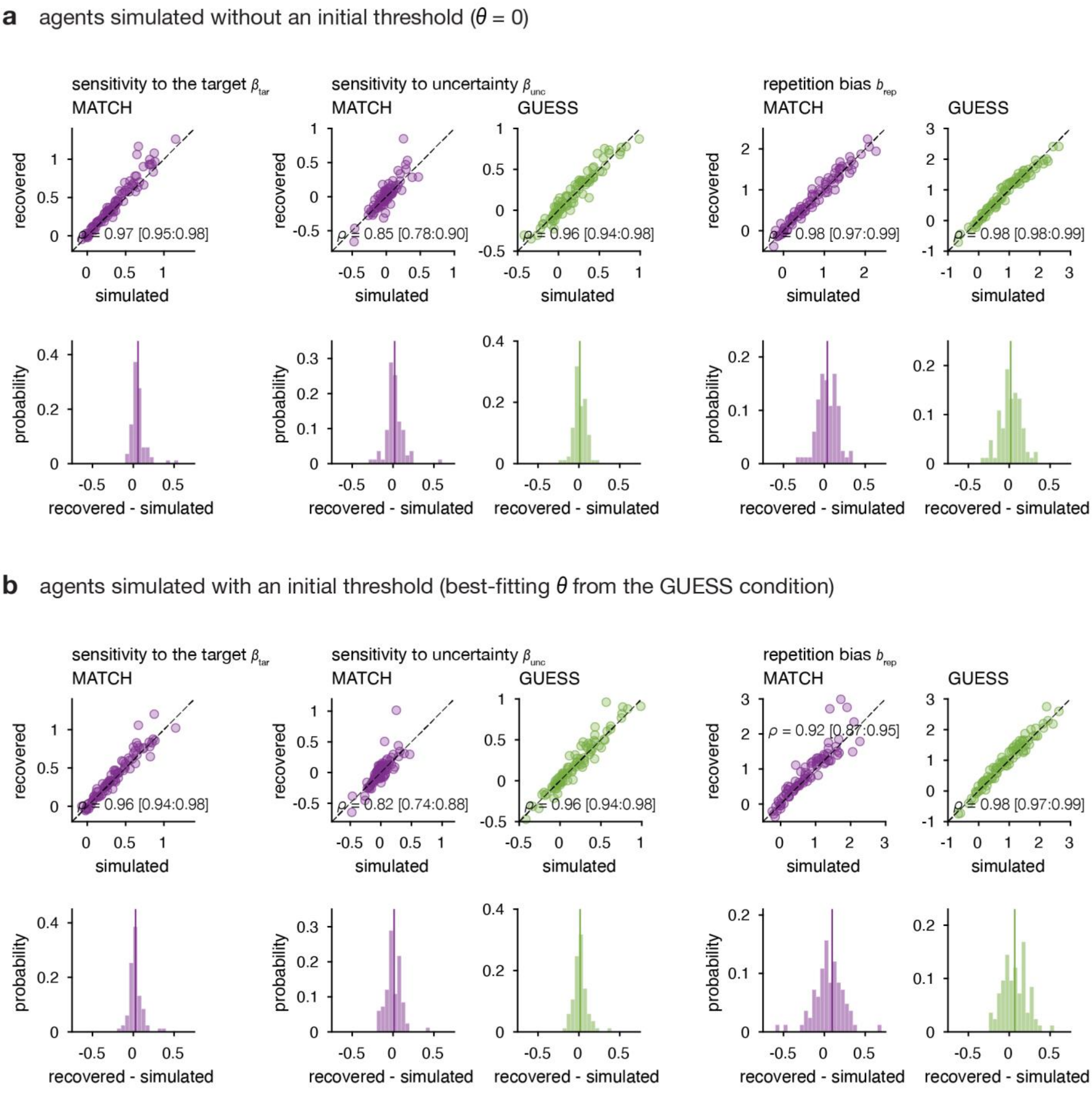

Supplementary Figure 6. Parameter recovery in a model with an initial threshold. a. Agents simulated without an initial threshold (θ = 0, *N* = 85 agents). Choices of agents without an initial sampling phase (θ set to 0), fitted using a model with an initial sampling phase (free θ). Top: correlation of the simulated and recovered parameters - sensitivity to the target (β_{tar}) in the MATCH condition, sensitivity to uncertainty (β_{unc}) in the MATCH and GUESS conditions, and repetition bias (b_{rep}) in the MATCH and GUESS conditions. Spearman's correlations, outliers further than three standard deviations from the mean were excluded. Bottom: distributions of the fitting errors (recovered – simulated values), the mean is displayed as a vertical line, outliers further than three standard deviations from the mean were excluded (*N* < 3 in all plots). **b. Agents simulated with an initial threshold (best-fitting θ from the GUESS condition, *N* = 85 agents).** Choices of agents with an initial sampling phase (θ set to the best-fitting values obtained for participants in the GUESS condition), fitted using a model with an initial sampling phase (free theta). Top: correlation of the simulated and recovered parameters - sensitivity to the target (β_{tar}) in the MATCH condition, sensitivity to uncertainty (β_{unc}) in the MATCH and GUESS conditions, and repetition bias (b_{rep}) in the MATCH and GUESS conditions. Spearman's correlations, outliers further than three standard deviations from the mean were excluded. Bottom: distributions of the fitting errors (recovered – simulated values), the mean is displayed as a vertical line, outliers further than three standard deviations from the mean were excluded (*N* < 3 in all plots).

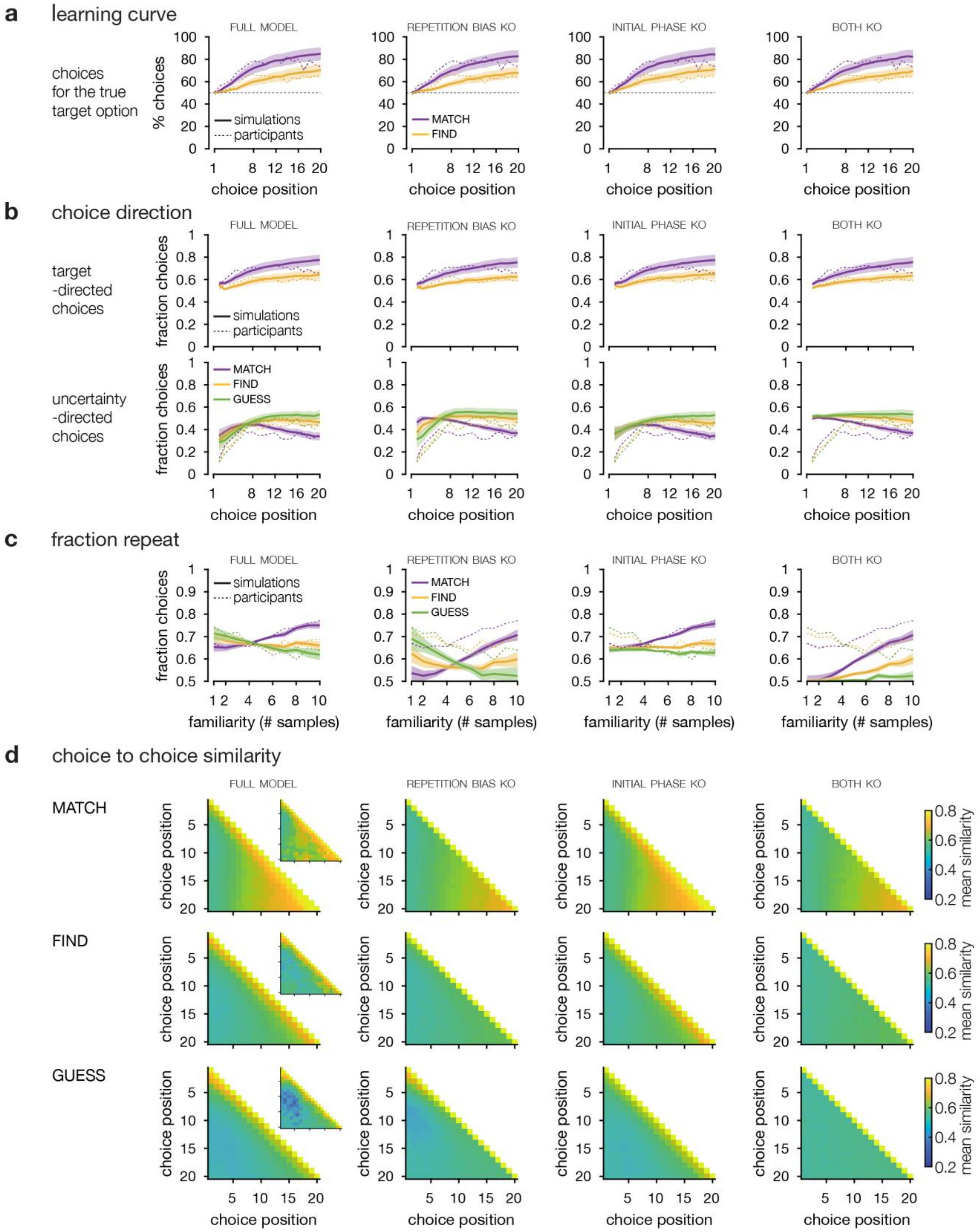
a
learning curve
choices for the true target option
% choices
FULL MODEL
REPETITION BIAS KO
INITIAL PHASE KO
BOTH KO
simulations
participants
MATCH
FIND
choice position
b
choice direction
target -directed choices
uncertainty -directed choices
fraction choices
GUESS
c
fraction repeat
familiarity (# samples)
d
choice to choice similarity
MATCH
FIND
GUESS
choice position
mean similarity

Supplementary Figure 7. Knock-out simulations in the third dataset (*N* = 31 participants). Columns different 'knock-out' versions of the model: the full version, with sensitivity to the target and to uncertainty, repetition bias and an initial sampling phase (first column), a model without the repetition bias (second column), a model without the initial repetitive phase (third column), and a model without initial sampling phase nor repetition bias (only sensitivity to the target colour and to uncertainty, last column). Choices of the different versions of the model were analysed through the same pipeline as the participants' choices. In purple for the MATCH condition, in green for the GUESS condition, and in yellow for the FIND condition; simulations in solid lines, participants in dotted lines for reference. **a. Learning curve.** Fraction of choices towards the option truly associated to the target colour, plotted against choice position for single-target sequences. Model simulations (solid lines, group means ± 95% CI), and participants' behaviour (dashed-lines). NB: in the FIND condition, this variable should not be interpreted as a performance index, as participants were explicitly informed that only their final choice, at the end of the sequence, contributed to their score. **b. Choice direction.** Top: in the MATCH and FIND conditions, fraction of target-directed choices at each choice in the sequence (solid lines: average of model simulations ± 95% CI, dashed lines: participants' average). For each choice, we defined the currently target option as the option with highest accumulated value in direction of the target colour category. Bottom: in all conditions, fraction of uncertainty-directed choices at each choice in the sequence (solid lines: average of model simulations ± 95% CI, dashed lines: participants' average). For each choice, we defined the uncertain option as the option whose current absolute accumulated value was the lowest. **c. Fraction repeat.** Fraction of repeat decisions on the following choice, as a function of how many times participants had already sampled this option earlier in the sequence (solid lines: simulations' average ± 95% within simulation CI, dashed lines: participants' average). **d. Choice to choice similarity.** For each condition, average similarity of each choice to the other choices in the same sequence, for model simulations (main triangles) and participants (inlays).

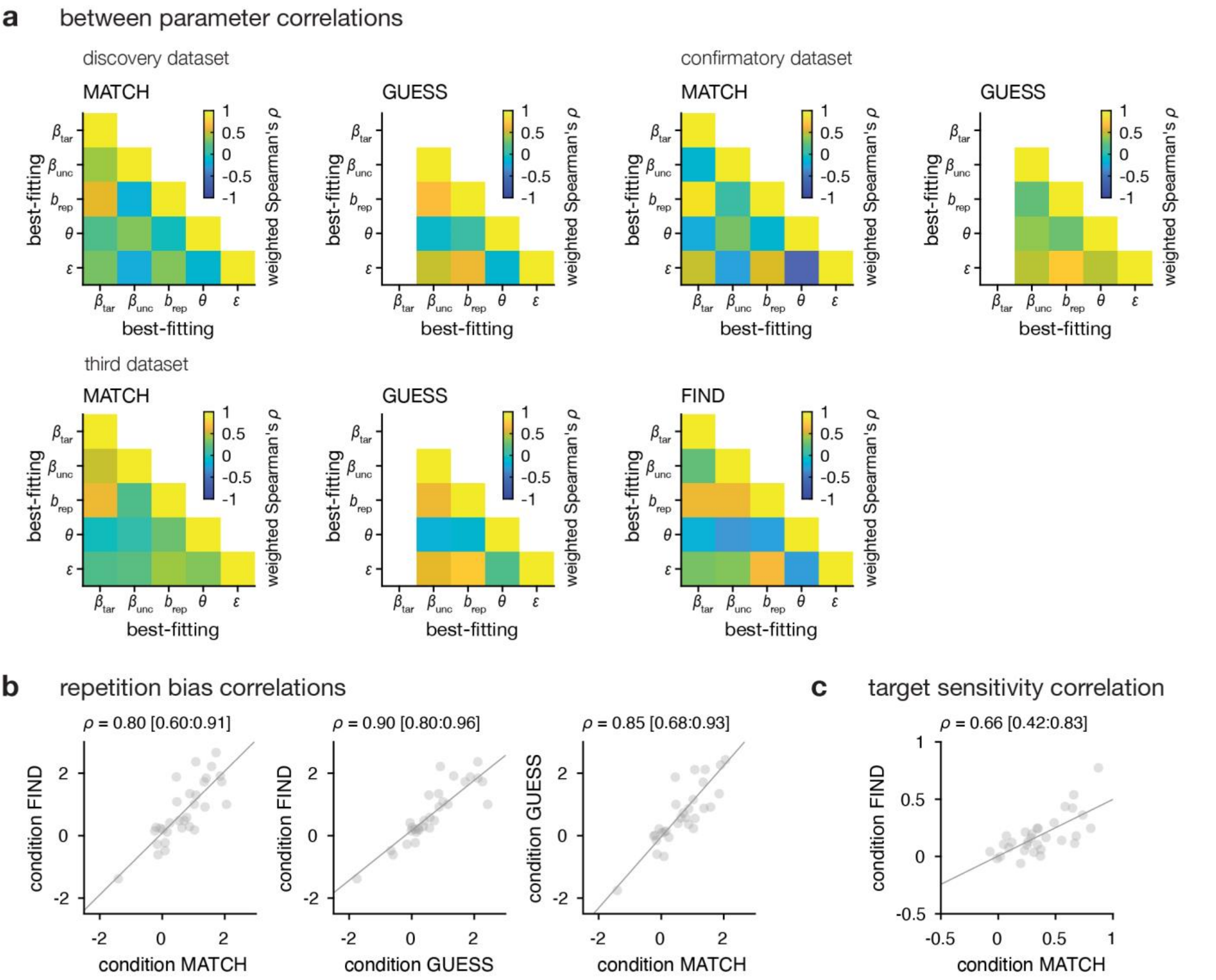

Supplementary Figure 8. Correlations in best-fitting parameters in the third dataset. a. Between parameter correlations. Correlation of best-fitting parameters obtained in each condition in all three datasets (Spearman's correlations, weighted by the goodness of fit of the model for each participant). Discovery dataset N = 27 participants, confirmatory dataset N = 27 participants, third dataset N = 31 participants. **b. Repetition bias correlations.** Correlation of repetition bias estimates obtained in the three conditions of the third dataset (Spearman's correlations, N = 31 participants.). **c. Target sensitivity correlation.** Correlation of target sensitivity estimates obtained in the MATCH and FIND conditions in the third dataset (the only conditions where participants were given a target in advance) - Spearman's correlation.

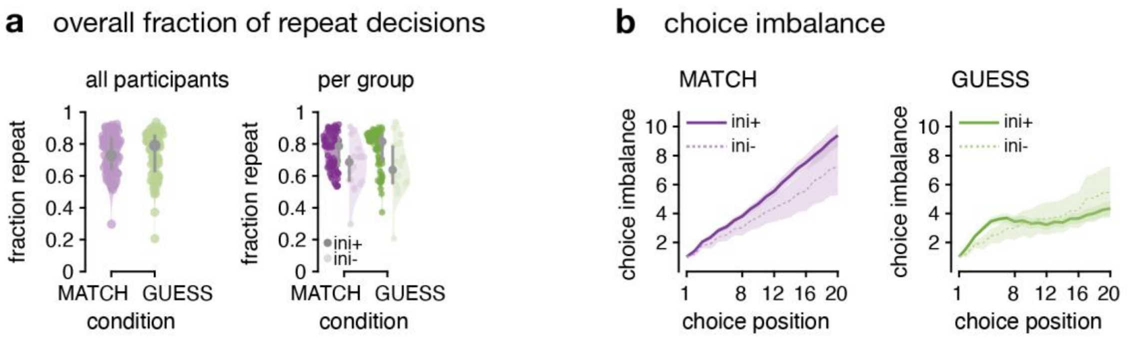

Supplementary Figure 9. Additional behavioural indices of the inter-individual differences in sampling patterns. The three datasets were pooled (total N = 85 participants) and participants were split in two groups based on whether a model with (ini+, N = 67 participants) or without (ini-, N = 18 participants) an initial sampling phase better accounted for their exploration patterns in GUESS sequences. GUESS condition in green and MATCH condition in purple. **a. Overall fraction of repeat decisions.** Fraction of repeat decisions in each condition, averaged per participant for all the participants (left, total N = 85), and for the ini+ and ini- participants separately (right, ini+ in full opacity, ini- group in transparency) – median ± inter-quartile range. **b. Choice imbalance.** Difference between the number of choices for the most and the least chosen option at each choice throughout the games. Solid lines display the average for participants whose best-fitting model in the GUESS condition included an initial sampling phase (ini+, N = 67), dotted lines show the average of participants whose best-fitting model did not include an initial sampling phase (ini-, N = 18) – group means ± 95% CI.

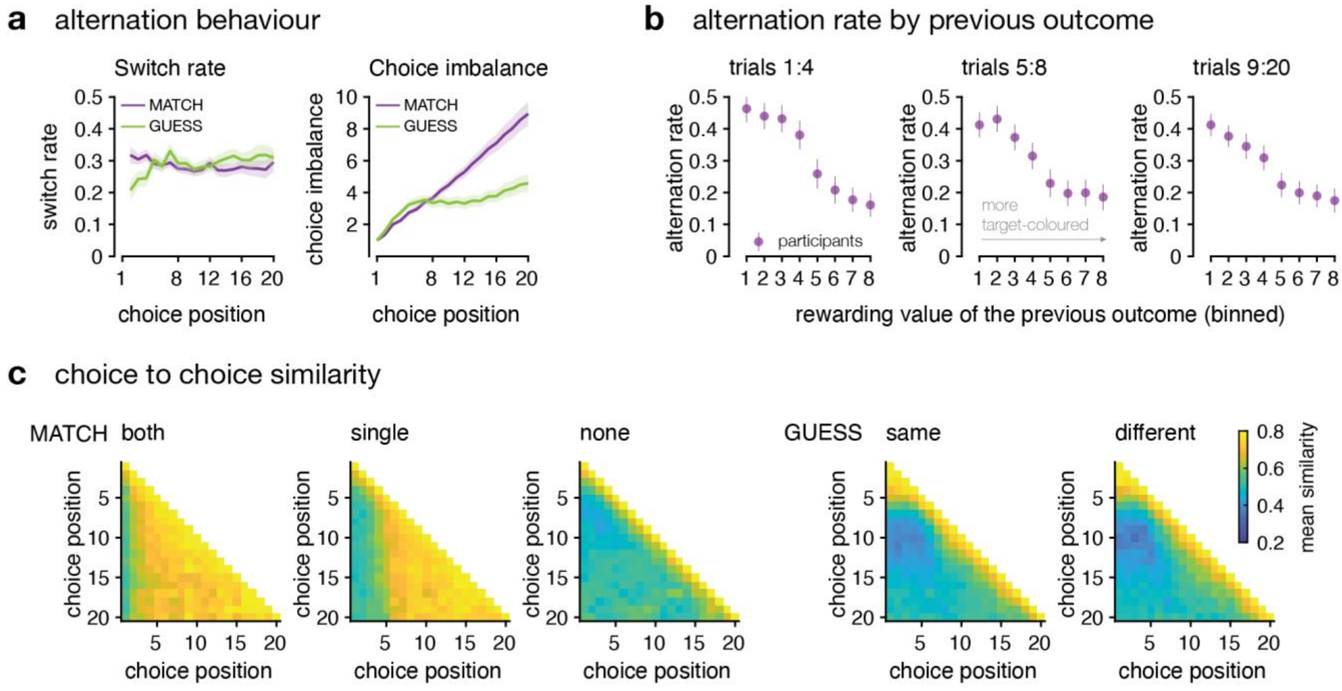

Supplementary Figure 10. Alternation behaviour in the initial choices. Figures display the pooled datasets (total N = 85 participants). MATCH condition in purple and GUESS condition in green. **a. Alternation behaviour.** Average switch rate at each choice position in the sequence (group mean ± 95% within participant CI). Average choice imbalance between the most and the least chosen option at each choice position in the sequence (group mean ± 95% CI) **b. Alternation rate by previous outcome.** In the MATCH condition, alternation rate in participants' decisions as a function of the rewarding value ("targetness" of the previous colour outcome) of the preceding outcome (binned into 8 bins), in the first four, following four, and remaining twelve choices. Group means (dots) and 95% CI (confidence bars). **c. Choice to choice similarity.** Average similarity of each choice to the other choices in the same sequence. In the MATCH condition, for sequences where a both, a single, or none of the options were associated to the target colour. In the GUESS condition, for sequences where the options were associated to the same, or different colours.

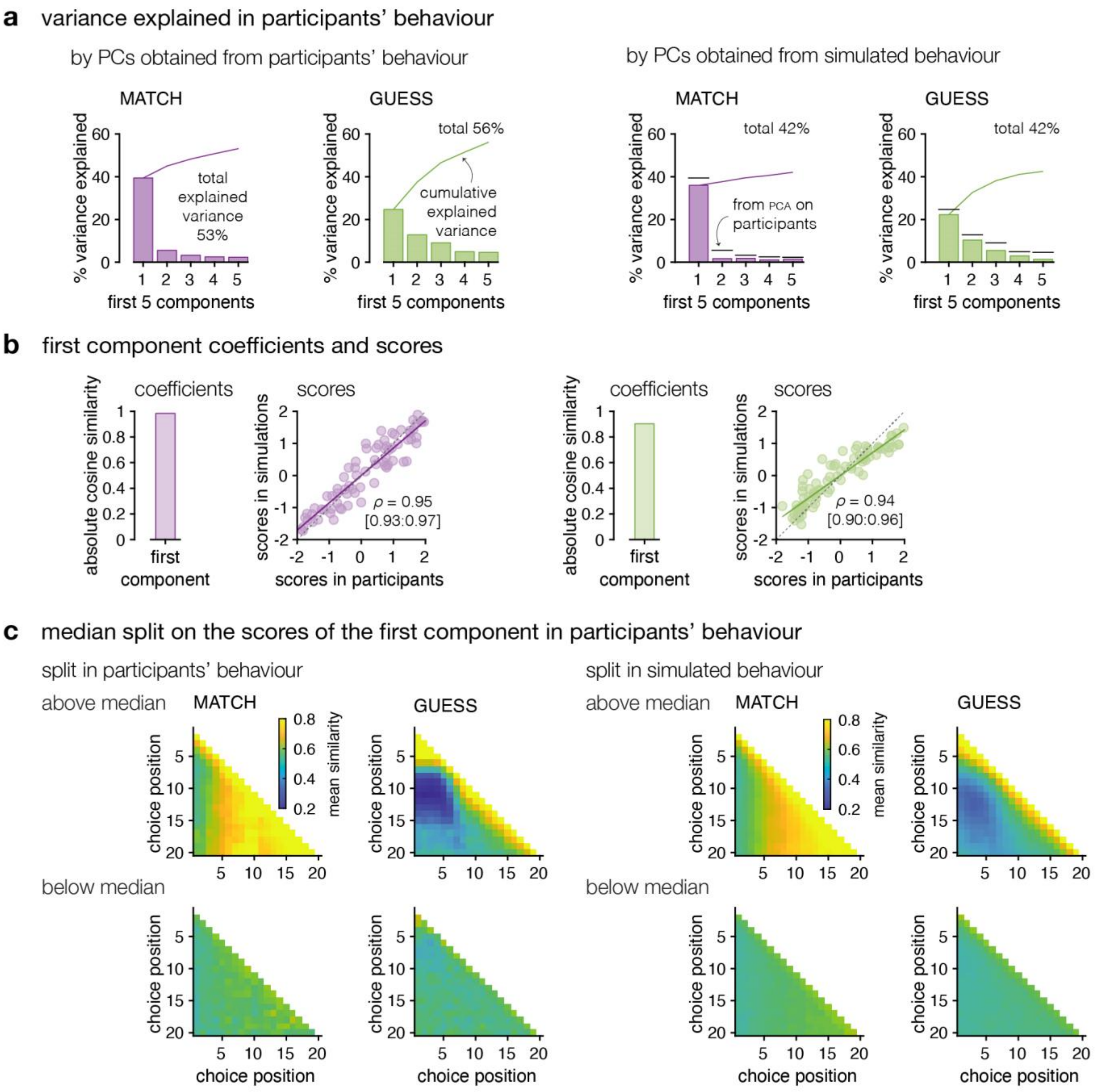

Supplementary Figure 11. Principal component analysis (PCA) of participants' behaviour vs. simulated behaviour. The three datasets were pooled for this analysis (total N = 85 participants). MATCH condition in purple and GUESS condition in green. **a. Variance explained in participants' behaviour**. Percentage of variance explained by the first five principal components (PCs) of the PCA on the participants' behaviour (left), and on the simulated behaviour (right), in the MATCH and the GUESS conditions. All three datasets were pooled for this analysis, using only the MATCH and GUESS conditions. The total variance explained by the first five principal components is annotated, plain line represents the cumulative explained variance, and horizontal black lines on the simulation plots (right) reproduce the variance explained in the participants' PCA. **b. First component coefficients and scores.** Comparison of the coefficients and scores of the first component of the PCA done on participants' behaviour and on simulated behaviour. Bar plots show the cosine similarity of the first component coefficients in each PCA. Scatter plots show the correlation of the individual scores (weights) in the first component of the participants PCAs and the simulations PCAs. Dotted lines are the identity line; solid lines are regression lines. Pearson's ρ correlation coefficients and bootstrapped 95% confidence intervals. **c. Median split on the scores of the first component in participants' behaviour.** Choice similarity matrices plotted for the participants' behaviour, for participants whose first PC was above and below the median (left). And for simulated behaviour based on the best-fitting parameter values of participants whose first PC scores were above or below the median (right).

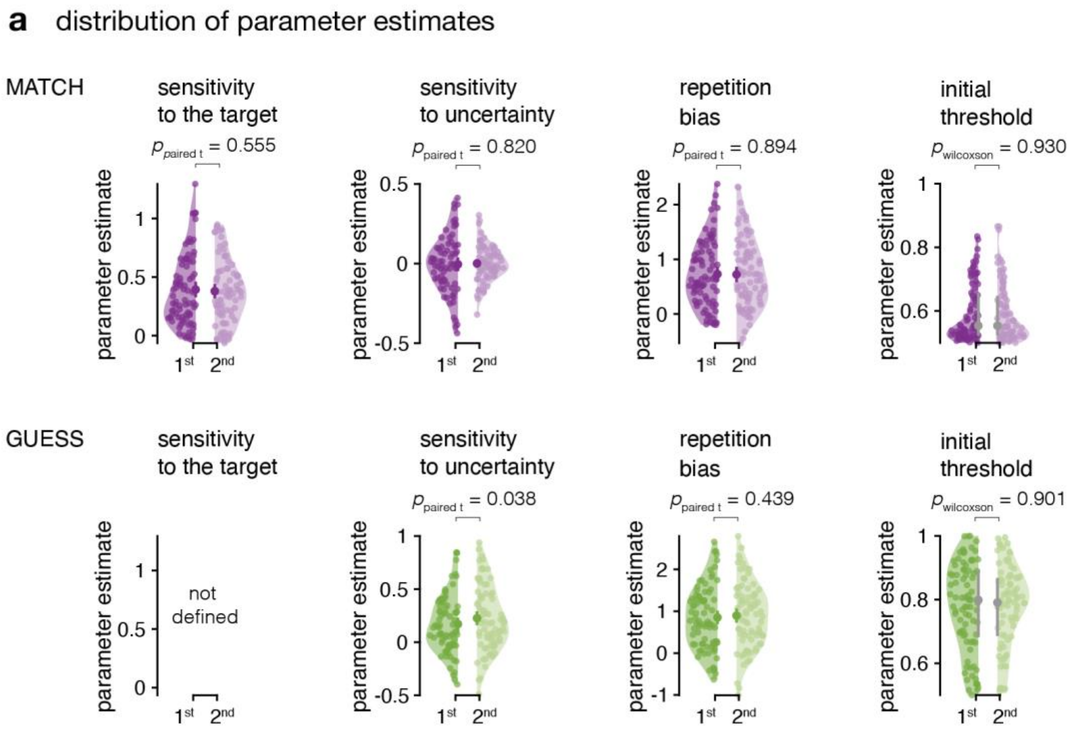

Supplementary Figure 12. Parameter reliability in the first v. second half of the task. The three datasets were pooled for this analysis (total N = 85 participants), using only the MATCH and GUESS conditions. **a. Distribution of parameter estimates.** For the MATCH (top row, in purple) and the GUESS condition (bottom row, in green): distribution of participant's parameter values estimated in the first v. the second half of the task – group means ± 95% CI plotted in colours, and group medians ± inter-quartile ranges for the bounded parameters plotted in grey. Data points outside of the first and third quartile by 1.5 inter-quartile ranges were considered outliers and excluded (Tuckey's method) – from left to right and top to bottom, N = 4, N = 15, N = 3, N = 7, not applicable, N = 3, N = 5, N = 0. The estimates from the two halves were compared with two-sided paired t-tests in unbounded parameters (first three) and with two-sided signed-rank tests for the initial threshold (bounded parameter).

Supplementary Tables

Performance		first dataset		second dataset		third dataset		
		MATCH	GUESS	MATCH	GUESS	MATCH	FIND	GUESS
	mean (s.d.)	76.16 (19.92)	78.94 (13.44)	74.65 (21.11)	78.36 (14.63)	78.23 (16,29)	89.72 (12.76)	86.69 (12.26)
	median (IQR)	81.25 (35.94)	81.25 (21.88)	84.38 (39.84)	81.25(15.62)	81.25 (31.25)	93.75 (12.50)	87.50 (12.50)
	z vs. chance	4.1	4.4	3.9	4.4	4.8	4.9	4.9
	p vs. chance			<0.001	<0.001	<0.001	<0.001	<0.001
	r	0.80	0.85	0.75	0.86	0.86	0.87	0.88
	95% CI on *r*	[0.60 : 0.90]	[0.69 : 0.93]	[0.51 : 0.88]	[0.71 : 0.93]	[0.73 : 0.93]	[0.75 : 0.94]	[0.76 : 0.94]

Supplementary table 1. Performance. In the MATCH condition, performance is defined as the percentage of target-oriented decisions on the last sample of the sequence. In the GUESS and FIND conditions, it is the percentage of correct responses at the final two-alternative forced choice. Note: In the FIND condition, the fraction of target-oriented choices at the last sampling decision is not directly a measure of performance (as participants were told they could freely sample and only the final question was worth points), but statistics are given here for comparison with the MATCH condition: mean (s.d.) = 63.91 (14.94), median (IQR) = 62.50 (25), *z* against chance = 4.0; $p < 0.001$; $r = 0.71$, 95% CI = [0.48 : 0.85]. s.d.: standard deviation, IQR: interquartile range, 95% CI: 95% confidence interval, *z* and *p* values from two-sided Wilcoxon signed-rank tests against chance (50%). First dataset $N = 27$ participants, second dataset $N = 27$ participants, third dataset $N = 31$ participants.

Overall fraction of repeat decisions	first dataset		second dataset		third dataset		
	MATCH	GUESS	MATCH	GUESS	MATCH	FIND	GUESS
mean (s.d.)	0.75 (0.10)	0.78 (0.10)	0.74 (0.13)	0.747 (0.134)	0.72 (0.15)	0.71 (0.17)	0.70 (0.17)
median (IQR)	0.78 (0.17)	0.82 (0.12)	0.72 (0.22)	0.79 (0.23)	0.72 (0.22)	0.71 (0.27)	0.70 (0.27)
z vs. chance	4.54	4.54	4.54	4.51	4.60	4.23	4.27
p vs. chance			<0.001	<0.001	<0.001	<0.001	0.001
r vs. chance	0.87	0.87	0.87	0.87	0.83	0.77	0.77
95% CI on *r*	[0.74 : 0.94]	[0.74 : 0.94]	[0.74 : 0.94]	[0.73 : 0.94]	[0.67 : 0.91]	[0.57 : 0.88]	[0.57 : 0.88]

Supplementary table 2. Overall fraction of repeat decisions. In all conditions, overall fraction of choices where participants repeated their previous choice. s.d.: standard deviation, IQR: interquartile range, CI: 95% confidence interval, *z* and *p* values from two-sided Wilcoxon signed-rank tests against chance (50%). First dataset *N* = 27 participants, second dataset *N* = 27 participants, third dataset *N* = 31 participants.

Fraction of repeat decisions ANOVA	first dataset			second dataset			third dataset		
	p(repeat) x condition	p(repeat) x prev. samples	p(repeat) x prev. samples x condition	p(repeat) x condition	p(repeat) x prev. samples	p(repeat) x prev. samples x condition	p(repeat) x condition	p(repeat) x prev. samples	p(repeat) x prev. samples x condition
F(d.f.)	0.08 (1, 26)	5.91 (9, 234)	24.49 (9, 234)	0.30 (1, 26)	1.47 (9, 234)	11.10 (9, 234)	4.38 (2, 60)	1.50 (2, 270)	6.17 (18, 540)
p				0.588	0.162	<0.001	0.017	0.148	<0.001
η_G^2	< 0.001	0.037	0.119	0.002	0.008	0.048	0.007	0.008	0.027

Supplementary table 3. Fraction of repeated decisions ANOVA. Repeated measures ANOVA for the fraction of repeat decisions as a function of the condition and of the number of samples previously taken from the same option. d.f.: degrees of freedom, η_G^2: partial eta squared. First dataset *N* = 27 participants, second dataset *N* = 27 participants, third dataset *N* = 31 participants.

Choice to choice similarity	first dataset				second dataset				third dataset					
	MATCH		GUESS		MATCH		GUESS		MATCH		FIND		GUESS	
	choices 1:4	1:4 to 8:11	choices 1:4	1:4 to 8:11	choices 1:4	1:4 to 8:11	choices 1:4	1:4 to 8:11	choices 1:4	1:4 to 8:11	choices 1:4	1:4 to 8:11	choices 1:4	1:4 to 8:11
Mean (s.d.)	0.65 (0.11)	0.56 (0.06)	0.80 (0.16)	0.35 (0.14)	0.65 (0.13)	0.57 (0.07)	0.74 (0.20)	0.42 (0.18)	0.63 (0.15)	0.54 (0.07)	0.68 (0.19)	0.47 (0.12)	0.71 (0.19)	0.42 (0.15)
Median (IQR)	0.65 (0.16)	0.55 (0.07)	0.88 (0.24)	0.40 (0.26)	0.64 (0.23)	0.56 (0.10)	0.86 (0.40)	0.45 (0.22)	0.60 (0.23)	0.54 (0.08)	0.69 (0.38)	0.49 (0.07)	0.69 (0.31)	0.47 (0.17)
z	4.38	3.95	4.38	-4.06	4.07	4.01	3.89	-2.26	4.03	2.92	3.95	-0.41	4.17	-2.65
p					<0.001	<0.001	<0.001	0.024	<0.001	0.004	<0.001	0.681	<0.001	0.008
r	0.84	0.76	0.84	-0.78	0.78	0.77	0.75	-0.43	0.72	0.52	0.71	-0.07	0.75	-0.48
95% CI	[0.68 : 0.93]	[0.54 : 0.88]	[0.68 : 0.93]	[-0.90 : -0.57]	[0.58 : 0.90]	[0.55 : 0.89]	[0.52 : 0.88]	[-0.70 : -0.07]	[0.50 : 0.86]	[0.21 : 0.74]	[0.47 : 0.85]	[-0.42 : 0.29]	[0.54 : 0.87]	[-0.71 : -0.15]

Supplementary table 4. Choice-to-choice similarity. Average similarity of choices 1:4, two-sided Wilcoxon signed-rank test against chance similarity (0.5). Average similarity of choices 1:4 to 8:11, two-sided Wilcoxon signed-rank tests against chance similarity (0.5). s.d.: standard deviation, IQR: interquartile range, *z*, *p* and *r* values from two-sided Wilcoxon signed-rank tests against chance similarity (0.5), 95% CI: 95% confidence interval on signed-rank *r* value. First dataset *N* = 27 participants, second dataset *N* = 27 participants, third dataset *N* = 31 participants.

Decision variable parameters		first dataset		second dataset		third dataset		
		MATCH	GUESS	MATCH	GUESS	MATCH	FIND	GUESS
Sensitivity to the target	mean	0.40	N.A.	0.40	N.A.	0.36	0.18	N.A.
	s.d.	0.27	N.A.	0.37	N.A.	0.25	0.18	N.A.
	median	0.36	N.A.	0.36	N.A.	0.34	0.14	N.A.
	IQR	0.39	N.A.	0.50	N.A.	0.37	0.18	N.A.
	t(d.f.)	7.6 (26)	N.A.	5.7 (26)	N.A.	7.9 (30)	5.8 (30)	N.A.
	p	N.A.	N.A.	<0.001	N.A.	<0.001	<0.001	N.A.
	d	1.47	N.A.	1.10	N.A.	1.43	1.03	N.A.
	CI	[0.91:2.01]	N.A.	[0.61:1.57]	N.A.	[0.92:1.92]	[0.59:1.47]	N.A.
Sensitivity to uncertainty	mean	-0.04	0.28	-0.01	0.18	0.04	0.12	0.12
	s.d.	0.14	0.20	0.22	0.34	0.15	0.26	0.25
	median	-0.02	0.28	-0.01	0.04	0.03	0.05	0.05
	IQR	0.13	0.27	0.12	0.43	0.22	0.33	0.25
	t(d.f.)	-1.4 (26)	7.5 (26)	-0.2 (26)	2.7 (26)	1.4 (30)	2.6 (30)	2.5 (30)
	p	N.A.	N.A.	0.875	0.013	0.082	0.008	0.008
	d	-0.28	1.43	-0.03	0.51	0.26	0.46	0.46
	95%CI	[-0.66:0.11]	[-0.89:1.97]	[-0.41:0.35]	[0.11:0.91]	[-0.10:0.61]	[0.08:0.83]	[0.08:0.82]
Repetition bias	mean	0.76	1.00	0.79	0.95	0.68	0.76	0.74
	s.d.	0.48	0.56	0.68	0.72	0.78	0.96	1.07
	median	0.70	1.07	0.58	0.90	0.70	0.48	0.57
	IQR	0.78	0.81	1.05	1.09	1.19	1.44	1.20
	t(d.f.)	8.2 (26)	9.2 (26)	6.0 (26)	6.9 (26)	4.9 (30)	4.4 (30)	3.9 (30)
	p	N.A.	N.A.	<0.001	<0.001	<0.001	<0.001	<0.001
	d	1.59	1.77	1.16	1.32	0.87	0.80	0.69
	95%CI	[1.01:2.15]	[1.16:2.37]	[0.66:1.64]	[0.79:1.83]	[0.45:1.28]	[0.39:1.20]	[0.29:1.08]

Supplementary table 5. Decision variable parameters. The parameters governing the decision variable were unbounded. In the second data set we tested them with two-sided *t*-tests against 0. In the third dataset, tests were one-sided *t*-tests against 0. s.d.: standard deviation, IQR: interquartile range, d.f.: degrees of freedom, *t* statistic from the *t*-tests, *p* value of the t-test, *d*: Cohen's *d*, and CI: 95% confidence interval on the effect size (*d*). First dataset *N* = 27 participants, second dataset *N* = 27 participants, third dataset *N* = 31 participants.

Initial sampling phase parameters		first dataset	second dataset	third dataset		
		MATCH vs. GUESS	MATCH vs. GUESS	MATCH vs. GUESS	FIND vs. GUESS	MATCH vs. FIND
Threshold level	mean	-0.20	-0.14	-0.21	-0.09	-0.12
	s. d.	0.18	0.21	0.17	0.20	0.19
	median	-0.26	-0.16	-0.25	-0.14	-0.10
	IQR	0.25	0.24	0.14	0.27	0.28
	z	-3.84	-3.17	-4.28	-2.52	-2.93
	p	N.A.	0.002	<0.001	0.006	0.002
	r	-0.74	-0.61	-0.77	-0.45	-0.53
	95% CI	[-0.87:-0.50]	[-0.80:-0.30]	[-0.88:-0.57]	[-0.70:-0.12]	[-0.74:-0.21]
Threshold hardness	mean	-0.11	0.07	-0.07	0.08	-0.15
	s. d.	0.25	0.23	0.37	0.18	0.39
	median	-0.02	0.06	0.00	0.03	-0.05
	IQR	0.25	0.15	0.36	0.11	0.38
	z	-1.6	1.69	-0.70	2.06	-1.43
	p	N.A.	0.091	0.481	0.040	0.153
	r	-0.31	0.33	-0.13	0.37	-0.26
	95% CI	[-0.62:0.07]	[-0.06:0.63]	[-0.46:0.24]	[0.02:0.64]	[-0.56:0.11]

Supplementary table 6. Initial sampling phase parameters. For plotting and statistical testing, the threshold level was transformed from logLR units to probabilities. Both parameters were thus bounded ([0.5:1] and [0:1]), we used non-parametric tests to compare conditions. For the second data set we used two-sided signed ranks tests between conditions. For the third dataset we used one-sided signed-ranks tests for the θ parameter and two-sided tests for the ε parameter because we had no directional predictions. s.d.: standard deviation, IQR: interquartile range, *z*: *z* statistic from the signed-rank tests, *p* value of the tests, CI: 95% confidence interval on the effect size (*r*). First dataset *N* = 27 participants, second dataset *N* = 27 participants, third dataset *N* = 31 participants.

Supplementary References

1. Lai, L. & Gershman, S. J. Policy compression: An information bottleneck in action selection. in *Psychology of Learning and Motivation* vol. 74 195–232 (Elsevier, 2021).
2. Gershman, S. J. Origin of perseveration in the trade-off between reward and complexity. *Cognition* **204**, 104394 (2020).
3. Gureckis, T. M. & Markant, D. B. Self-Directed Learning: A Cognitive and Computational Perspective. *Perspect Psychol Sci* **7**, 464–481 (2012).
4. Markant, D. B. & Gureckis, T. M. Is it better to select or to receive? Learning via active and passive hypothesis testing. *Journal of Experimental Psychology: General* **143**, 94–122 (2014).
5. Restle, F. THE SELECTION OF STRATEGIES IN CUE LEARNING. *Psychological Review* **69.4**, 329 (1962).
6. Bower, G. & Trabasso, T. REVERSALS PRIOR TO SOLUTION IN CONCEPT IDENTIFICATION. *Journal of Experimental Psychology* **66.4**, 409 (1963).
7. Bonawitz, E., Denison, S., Gopnik, A. & Griffiths, T. L. Win-Stay, Lose-Sample: A simple sequential algorithm for approximating Bayesian inference. *Cognitive Psychology* **74**, 35–65 (2014).
