# Supplementary material for: Competing cognitive pressures on human exploration in the absence of trade-off with exploitation
Source: Nat Commun. 2026 Feb 12;17:883. doi: 10.1038/s41467-026-68639-2 (PMC12901306; doi:10.1038/s41467-026-68639-2)
Supplement: Supplementary file 2 — Reporting Summary [file 41467_2026_68639_MOESM2_ESM.pdf]

nature portfolio

Corresponding author(s): Clemence Almeras & Valentin Wyart

Last updated by author(s): 2025/12/10

Reporting Summary

Nature Portfolio wishes to improve the reproducibility of the work that we publish. This form provides structure for consistency and transparency in reporting. For further information on Nature Portfolio policies, see our Editorial Policies and the Editorial Policy Checklist.

Statistics

For all statistical analyses, confirm that the following items are present in the figure legend, table legend, main text, or Methods section.

n/a	Confirmed	
☐	☒	The exact sample size (*n*) for each experimental group/condition, given as a discrete number and unit of measurement
☐	☒	A statement on whether measurements were taken from distinct samples or whether the same sample was measured repeatedly
☐	☒	The statistical test(s) used AND whether they are one- or two-sided<br>*Only common tests should be described solely by name; describe more complex techniques in the Methods section.*
☐	☒	A description of all covariates tested
☐	☒	A description of any assumptions or corrections, such as tests of normality and adjustment for multiple comparisons
☐	☒	A full description of the statistical parameters including central tendency (e.g. means) or other basic estimates (e.g. regression coefficient) AND variation (e.g. standard deviation) or associated estimates of uncertainty (e.g. confidence intervals)
☐	☒	For null hypothesis testing, the test statistic (e.g. *F*, *t*, *r*) with confidence intervals, effect sizes, degrees of freedom and *P* value noted<br>*Give P values as exact values whenever suitable.*
☒	☐	For Bayesian analysis, information on the choice of priors and Markov chain Monte Carlo settings
☒	☐	For hierarchical and complex designs, identification of the appropriate level for tests and full reporting of outcomes
☐	☒	Estimates of effect sizes (e.g. Cohen's *d*, Pearson's *r*), indicating how they were calculated

Our web collection on statistics for biologists contains articles on many of the points above.

Software and code

Policy information about availability of computer code

Data collection	The code used to run the experiments described in the study and the behavioural data is available at https://gitlab.com/cle-a/colpub ; https://doi.org/10.5281/zenodo.18215678).<br>The task was run using the Psychtoolbox-3 toolbox (version 3.0.14) for MATLAB 2017b (The Mathworks).
Data analysis	The main analysis pipeline is available on a public repository (https://gitlab.com/cle-a/colpub ; https://doi.org/10.5281/zenodo.18215678).<br>The main analysis pipeline has been uploaded prior to the collection of the replication dataset on a dedicated repository (https://gitlab.com/cle-a/colrep). The model fitting code has been updated during the analysis of the review process, and the final version is available on the main repository.<br>For model fitting we used the fmincon function from Matlab 2017b's Optimization toolbox, and the BADS (Bayesian Adaptive Direct Search) toolbox v1.0.8, and we used SPM12 version 6906 for BMS (Bayesian model selection). The analysis code also uses Matlab's Image processing toolbox (2017b), and Laurent Caplette's simple_mixed_anova function (see code repository for link).

For manuscripts utilizing custom algorithms or software that are central to the research but not yet described in published literature, software must be made available to editors and reviewers. We strongly encourage code deposition in a community repository (e.g. GitHub). See the Nature Portfolio guidelines for submitting code & software for further information.

Data

Policy information about availability of data

All manuscripts must include a data availability statement. This statement should provide the following information, where applicable:

- Accession codes, unique identifiers, or web links for publicly available datasets
- A description of any restrictions on data availability
- For clinical datasets or third party data, please ensure that the statement adheres to our policy

The data used in this study (raw anonymised behavioural data and summary tables) are available at https://gitlab.com/cle-a/colpub, DOI: 10.1038/s41562-022-01445-0

Research involving human participants, their data, or biological material

Policy information about studies with human participants or human data. See also policy information about sex, gender (identity/presentation), and sexual orientation and race, ethnicity and racism.

Reporting on sex and gender	We did not plan analyses based on sex or gender for this dataset. We had no theoretically motivated hypothesis for doing so. We included participants to achieve roughly balanced samples based on self-reported sex - overall numbers are reported below. Consent was obtained from tested participants for sharing individual, anonymized task data, not self-reported sex.
Reporting on race, ethnicity, or other socially relevant groupings	We did not collect data on race, ethnicity or other socially relevant grouping variables.
Population characteristics	The sample is a convenience sample. We recruited through local or national mailing list advertising studies to an audience composed mainly of students. This includes mostly young WEIRD participants. Further research should look into the determinants of inter-individual variability in participants' tendency to chunk samples and how WEIRD-ness of the population may impact the results we describe.<br><br>We asked for participants aged between 18 and 35 years, right-handed, with normal or correct-to-normal vision, and had no history of psychiatric or neurological disorder (including claustrophobia).<br><br>We collected the following data:<br>Dataset 1: N = 30, 15 females, mean age = 25 years (standard deviation = 4.3).<br>Dataset 2: N = 30, 15 females, mean age = 25 years (standard deviation = 3.7).<br>Dataset 3: N = 38, 21 females, mean age 24 years (standard deviation = 4.4).<br>Some data was subsequently excluded (see below).
Recruitment	For the first dataset, participants were recruited through a local, open mailing list advertising psychology and neuroscience experiments in the Paris area.<br>For the second dataset, we used the same mailing list, and a national website advertising short-term jobs for adult students (L'Etudiant).<br>For the third dataset, participants were recruited exclusively through the website L'Etudiant.<br><br>Participants recruited through the psychology mailing list are typically students (in the age range we targeted), and typically accustomed to psychology experiments. Participants recruited through L'Etudiant were also a majority of students, but they had never taken part in psychology studies.
Ethics oversight	Ethics approval was obtained from the relevant authorities covering all three datasets (Comité de Protection des Personnes Ile-de-France VI, ID RCB: 2007-A01125-48, 2017-A01778-45). All participants gave written informed consent before taking part in the study.

Note that full information on the approval of the study protocol must also be provided in the manuscript.

Field-specific reporting

Please select the one below that is the best fit for your research. If you are not sure, read the appropriate sections before making your selection.

☐ Life sciences ☒ Behavioural & social sciences ☐ Ecological, evolutionary & environmental sciences

For a reference copy of the document with all sections, see nature.com/documents/nr-reporting-summary-flat.pdf

Behavioural & social sciences study design

All studies must disclose on these points even when the disclosure is negative.

Study description	Across three independent datasets, participants were asked to play sequences of two-alternative forced choices. The study aimed to qualitatively and quantitatively characterise the sampling patterns of human participants in the presence vs. in the absence of immediate rewards after each decision.
Research sample	After exclusion (see below), the samples were:<br><br>- Dataset 1: final N = 27, 12 females, mean age 25 (sd 4)<br>- Dataset 2: final N = 27, 13 females mean age 25 (sd 4)<br>- Dataset 3: final N = 31, 16 females, mean age 24 (sd 4)<br><br>Recruitment criteria were: age between 18 and 35 years, right-handed, with normal or correct-to-normal vision, and no history of psychiatric or neurological disorder (including claustrophobia). The exclusion criteria are detailed below.<br><br>The sample was not designed to be representative of the general population.
Sampling strategy	See above, the sample was a convenience sample. Because we had no specific expectation regarding effect sizes, sample size was determined to match or exceed earlier studies of human exploration patterns in the literature. For the third dataset, there were fewer trials per condition per participant. We balanced this by aiming to reach N = 30 adult participants after exclusion.
Data collection	The task was run using the Psychtoolbox-3 toolbox (version 3.0.14) for MATLAB 2017b (The Mathworks). The testing conditions were slightly different across datasets.<br>Dataset 1: eye position was recorded during the task using an EyeLink-1000 Plus eye-tracker system (SR Research), and participants' head movements were constrained by a chin rest.<br>Dataset 2 and 3: Participants were seating freely, with their eyes at approximately 70 cm from a 24-inch LCD screen with a resolution of 1,920 × 1,080 pixels and a refresh rate of 60 Hz.<br>The participants were left alone in the soundproof booth while they completed the task, the experimenter only entered the room between blocks or on participant request (which did not occur). No one else was present than the participant.<br>Because the study is a within participant design, all participants were doing all conditions and no blinding was involved.
Timing	Dataset 1: 27 Nov. to 18 Dec. 2018<br>Dataset 2: 7-15 Oct. 2019<br>Dataset 3: 5-16 Oct. 2020
Data exclusions	We report that we excluded participants whose sampling strategy was stereotyped or did not depend on presented outcomes. We computed the number of stereotyped sequences for each participant: sequences where the participant systematically alternated between left and right responses, or between the two options, and sequences where the participant sampled a single option throughout the whole sequence. We excluded participants who produced more than 50% of such stereotyped sequences in any condition.<br><br>Dataset 1: N = 3; final N = 27, 12 females, mean age 25 (sd 4)<br>Dataset 2: N = 3; final N = 27, 13 females mean age 25 (sd 4)<br>Dataset 3: N = 6; final N = 31, 16 females, mean age 24 (sd 4)
Non-participation	In the third dataset, one participant left before completing the study due to COVID-19 symptoms.
Randomization	We used a within subject design: Each participant performed all the conditions. The conditions were defined by giving different instructions to participants: By definition participants were not blind to conditions, nor were the experimenters.

Reporting for specific materials, systems and methods

We require information from authors about some types of materials, experimental systems and methods used in many studies. Here, indicate whether each material, system or method listed is relevant to your study. If you are not sure if a list item applies to your research, read the appropriate section before selecting a response.

Materials & experimental systems

n/a	Involved in the study
☒	☐ Antibodies
☒	☐ Eukaryotic cell lines
☒	☐ Palaeontology and archaeology
☒	☐ Animals and other organisms
☒	☐ Clinical data
☒	☐ Dual use research of concern
☒	☐ Plants

Methods

n/a	Involved in the study
☒	☐ ChIP-seq
☒	☐ Flow cytometry
☒	☐ MRI-based neuroimaging

Plants

Seed stocks	Not applicable.
Novel plant genotypes	Not applicable.
Authentication	Not applicable.
